# Supplementary figures and images for: Co-occurrence patterns of litter decomposing communities in mangroves indicate a robust community resistant to disturbances
Source: PeerJ. 2018 Oct 4;6:e5710. doi: 10.7717/peerj.5710 (PMC6174875; doi:10.7717/peerj.5710)

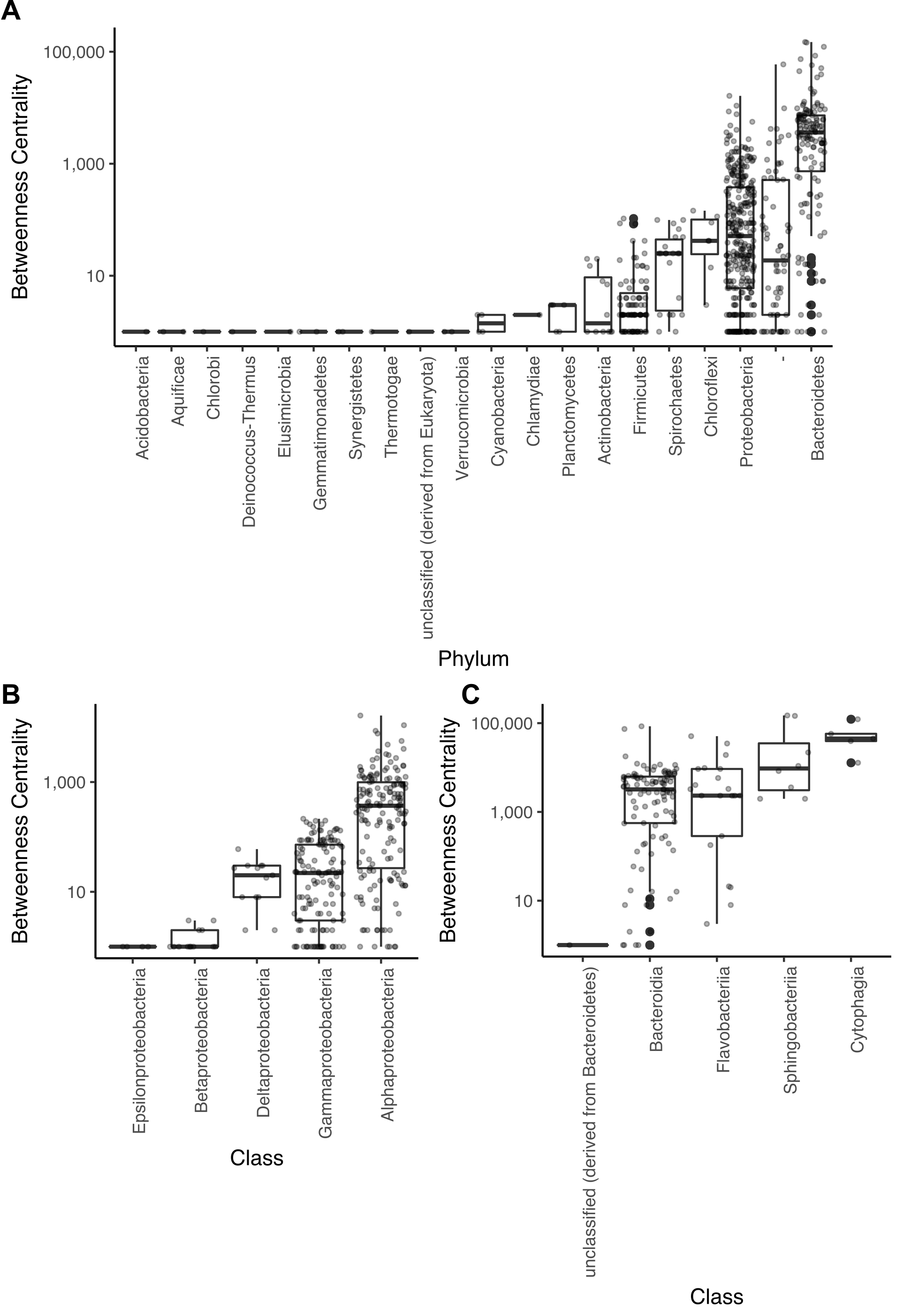

Supplement: Figure S2 — Jitter gray dots represent each node (BwC). Black dots represent boxplot outliers. A, Individual nodes BwC separated by phylum; B, Proteobacteria BwC separated by class; C, Bacteroidetes BwC separated by class. [file peerj-06-5710-s002.png]

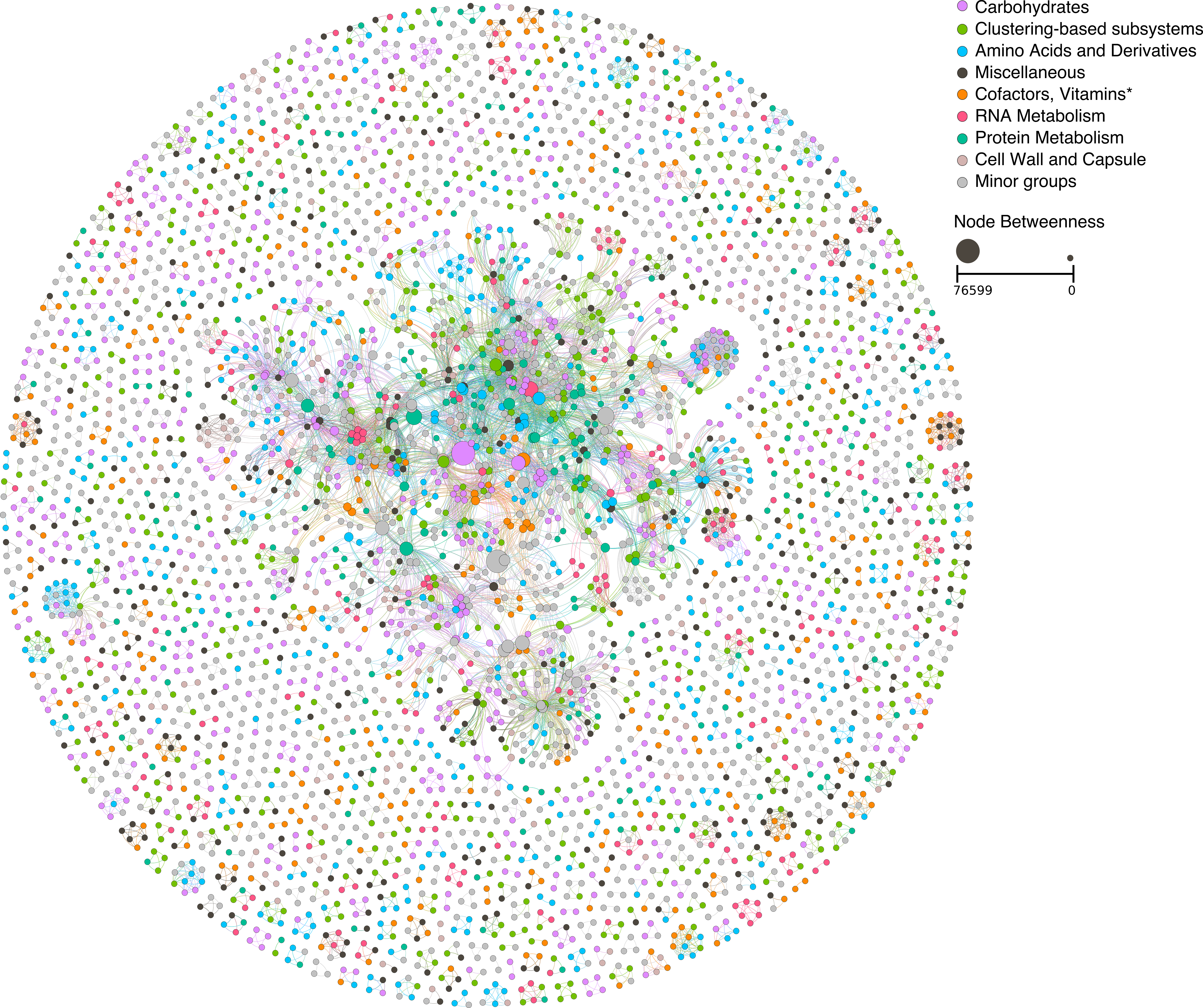

Supplement: Figure S3 — Node size is proportional to the Edge Betweenness. [file peerj-06-5710-s003.png]

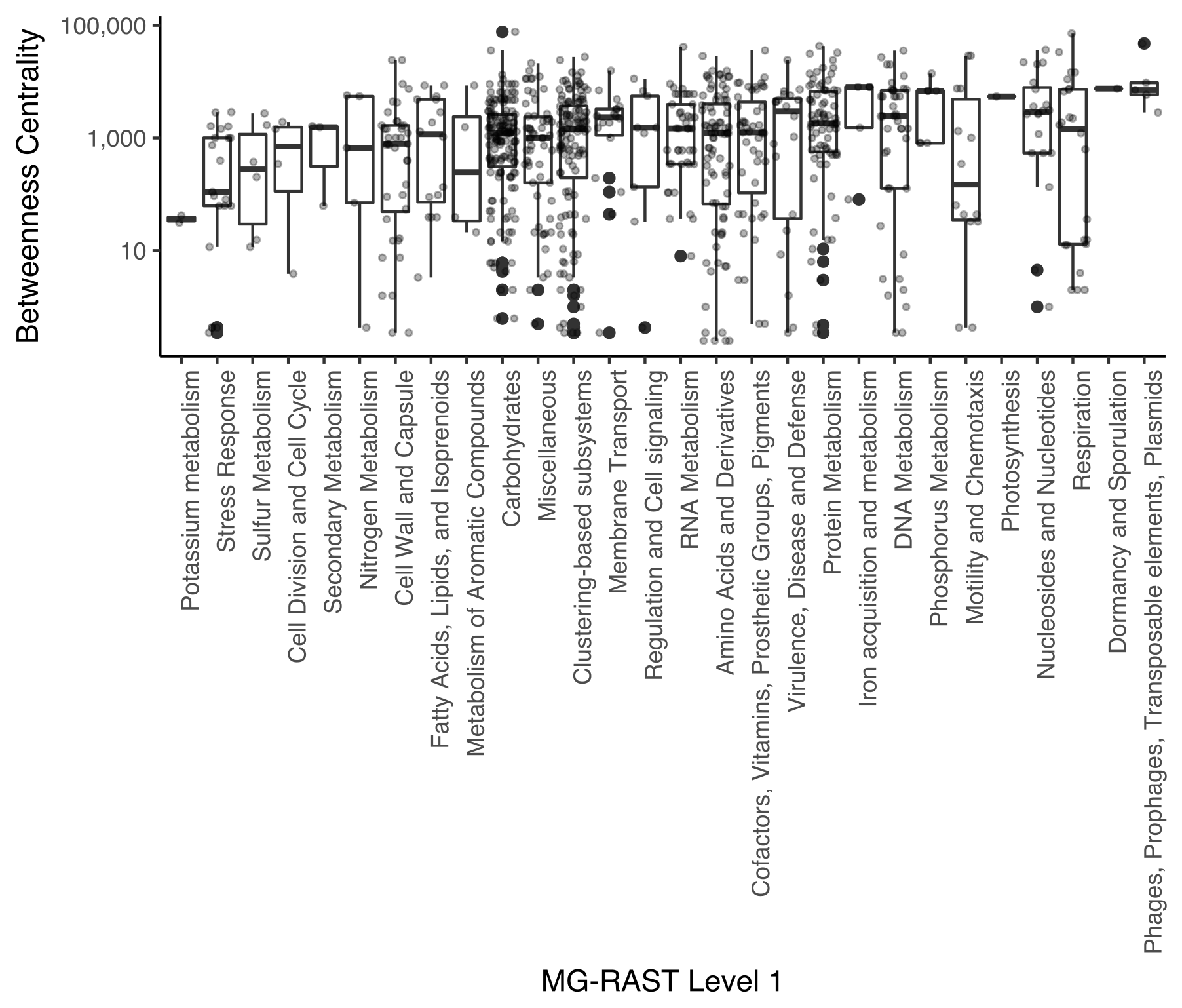

Supplement: Figure S4 — Jitter gray dots represent each node (BwC). Black dots represent boxplot outliers. [file peerj-06-5710-s004.png]
